# High electrolyte uptake of MXene integrated membrane separators for Zn-ion batteries

**DOI:** 10.1038/s41598-022-24578-8

**Published:** 2022-11-19

**Authors:** Chutiwat Likitaporn, Manunya Okhawilai, Pornnapa Kasemsiri, Jiaqian Qin, Pranut Potiyaraj, Hiroshi Uyama

**Affiliations:** 1 Nanoscience and Technology Interdisciplinary Program, Graduate School, Chulalongkorn University, Bangkok, 10330 Thailand; 2 Metallurgy and Materials Science Research Institute, Chulalongkorn University, Bangkok, 10330 Thailand; 3 Center of Excellence in Responsive Wearable Materials, Chulalongkorn University, Bangkok, 10330 Thailand; 4 Department of Chemical Engineering, Faculty of Engineering, Sustainable Infrastructure Research and Development Center, Khon Kaen University, Khon Kaen, 40002 Thailand; 5 Department of Materials Science, Faculty of Science, Chulalongkorn University, Bangkok, 10330 Thailand; 6 Department of Applied Chemistry, Graduate School of Engineering, Osaka University, Osaka, 565-0871 Japan

**Keywords:** Porous materials, Batteries, Materials for energy and catalysis, Batteries

## Abstract

The recent development of separators with high flexibility, high electrolyte uptake, and ionic conductivity for batteries have gained considerable attention. However, studies on composite separators with the aforementioned properties for aqueous electrolytes in Zn-ion batteries are limited. In this research, a polyacrylonitrile (PAN)/bio-based polyurethane (PU)/Ti_3_C_2_T_x_ MXene composite membrane was fabricated using an electrospinning technique. Ti_3_C_2_ MXene was embedded in fibers and formed a spindle-like structure. With Ti_3_C_2_T_x_ MXene, the electrolyte uptake and ionic conductivity reached the superior values of 2214% and 3.35 × 10^−3^ S cm^−1^, respectively. The composite membrane presented an excellent charge–discharge stability when assembled in a Zn//Zn symmetrical battery. Moreover, the developed separator exhibited a high flexibility and no dimensional and structural changes after heat treatment, which resulted in the high-performance separator for the Zn-ion battery. Overall, the PAN/bio-based PU/Ti_3_C_2_T_x_ MXene composite membrane can be potentially used as a high-performance separator for Zn-ion batteries.

## Introduction

Flexible electronic devices have recently gained broad interest from the academic and industrial sectors. However, flexible batteries are required to power such devices. Separators are noteworthy porous membrane in batteries; they separate the two electrodes, which may cause the short circuit of battery cell if they come into contact with each other, and allow the penetrating movement of ionic charges. The mechanism of ion movement determines the battery performance. To enhance ionic conductivity, scientists should develop the most crucial property of separators, that is, the electrolyte uptake value. The high porosity of materials allows the effective uptake and retention of electrolytes in the separator, providing a long operating life to batteries and reducing the internal ionic resistance of battery cells^1^. The electrospinning technique produces an outstandingly high-porosity fiber membrane, high surface-area-to-volume ratio, and micron- to nanosized fiber diameters^2^. Various types of polymer precursor-based electrospun separators have been studied; examples include polyvinylidene fluoride (PVDF)^3,4^, poly(vinylidene fluoride-co-hexafluoropropylene)^5^, polyimide (PI)^6^, polyacrylonitrile (PAN)^7^, and polymer-modified glass fiber membranes^8^. In these polymer precursors, polar groups serve as coordinating sites for cation movement^9^. A separator based on PAN/bio-based polyurethane (PU) electrospun membranes with a high ionic conductivity has been developed by Saisangtham S. et al.^10^. The parameters that affect membrane properties, including polymer concentration, applied voltage, and distance from the tip to collector, were observed. According to the Taguchi design of the experiment and optimization using Grey relational analysis, the membrane produced from 14 wt% polymer concentration, 25 kV applied voltage, and 16 cm distance from the tip to the collector exhibited an excellent electrolyte uptake of 1,971% and conductivity of 3.11 mS cm^−1^. The PI electrospun separator provided 2522% Li electrolyte uptake and a porosity of 92%^6^. Notably, higher C-rate capability, better cycling performance, and lower cell resistance have been reported with the use of electrospun membranes^11^. The preparation of composite separators filled with porous inorganic particles, for example, alumina (Al_2_O_3_)^12^, silica (SiO_2_)^13^, zirconia (ZrO_2_)^12^, and titanium dioxide (TiO_2_)^14^, is a key technique to enhancing the electrolyte uptake. Moreover, inorganic particles develop the mechanical strength and thermal resistance of the resulting composite separators. A membrane based on SiO_2_/PVDF was developed^11^. The porosity of 24 wt% SiO_2_ in the PVDF composite accounted for 120% enhancement from the pure PVDF membrane, resulting in 128% and 152% increments in electrolyte uptake and ionic conductivity, respectively. In addition, other filler structures are incorporated into separator materials to enhance their performance; these structures include high-porosity materials such as metal organic frameworks (MOFs)^15^ and two-dimensional (2D) structure as MXene^16^. Especially, MXene probably increases the separator performance due to its hydrophilic nature.

MXene is a 2D structure combining transition metal carbides and carbonitrides. Typically, MXene is synthesized from the ${M}_{n+1}A{X}_{n}$ phase (MAX phase), where M is a transition metal, A refers to a IIIA or IVA element, X represents carbon or nitrogen atoms, and n = 1, 2, 3 integers. Therefore, different MXene chemical structures, including Ti_3_C_2_T_x_^17–19^, Ta_4_C_3_^20,21^, Nb_2_C^22–24^, and ZrC^25,26^, are available. Ti_3_C_2_ has been used widely. MXene exhibits excellent inherent physicochemical properties, good mechanical strength, and electrical and thermal conductivities and thus has attracted considerable attention from the academic sectors givens its broad applications, especially in high-conductivity materials, including the electrode for supercapacitors^27,28^, promoters for catalysts, and absorbents for heavy-metal ions. In general, delaminated MXene is used in such applications. To further exploit the unique properties of MXene, we developed an MXene microsheet composite membrane in this research. With the abundant polar group in the particle, MXene flakes are compatible with the polymer matrix, which is an efficient way to obtain multifunctional polymer composites. Moreover, MXene exhibits hydrophilicity and is expected to absorb and retain aqueous electrolyte in battery cells, thus enhancing their performance^29^.

Herein, a composite separator membrane based on PAN/bio-based PU filled with Ti_3_C_2_T_x_ MXene was fabricated using an electrospinning technique. The suitable processing conditions for PAN/bio-based PU/Ti_3_C_2_T_x_ MXene were studied to obtain continuous fibers with well-dispersed Ti_3_C_2_ MXene embedded in the fiber structure. The effects of Ti_3_C_2_ MXene content on morphological properties, chemical interaction, and mechanical strength of the developed composite membrane were investigated. Various types of membranes have been developed for use in Li-ion batteries. However, very limited publications have focused on separator membranes suitable for Zn-ion batteries. The developed PAN/bio-based PU/Ti_3_C_2_T_x_ MXene membrane will be applied to aqueous-based electrolytes in battery systems, which are a safe, economically, and eco-friendly system, especially Zn-ion batteries.

## Materials and Methods

### Materials

PAN with an average molecular weight of 150,000 g mol^−1^, dibutyltin dilaurate (95%), and zinc trifluoromethanesulfonate (98%) were purchased from Sigma-Aldrich Corporation (USA). Polycaprolactone diol having an average Mn of 2000 g mol^−1^ was purchased from Sigma-Aldrich Corporation (China). Ethylene glycol (Grade AR) was purchased from QReC (New Zealand). Dimethylformamide (DMF) was supplied by RCI Labscan limited (Thailand). Partially bio-based diisocyanate (Tolonate™ X FLO 100) was kindly provided by Vencorex Co. Ltd (France). Its chemical structure is shown in Figure S1(a), and the synthesized bio-based PU is presented in Figure S1(b). Ti_3_C_2_T_x_ MXene with a size > 37 µm and purity > 98% was purchased from Wuxi Admas Technology Co., Ltd, Jiangsu, China.

### Synthesis of bio-based PU

In this study, bio-based PU was synthesized using polycaprolactone diol, partially bio-based diisocyanate (Figure S1(a)), and ethylene glycol at a molar ratio of 2.1:1:1 in a four-necked round-bottom flask equipped with a mechanical stirrer, thermometer, and condenser, following a previously described method^10^. All reactants were charged in the reactor, which contained DMF solvent with the addition of dibutyltin dilaurate catalyst. The reaction was carried out at a temperature of 70 °C under N_2_ atmosphere. The as-synthesized bio-based PU was purified in a large amount of ethanol. Then, bio-based PU was used to evaporate ethanol. Figure S1(b) shows the chemical structure of bio-based PU.

### Electrospinning of PAN/bio-based PU

**Figure 1:**
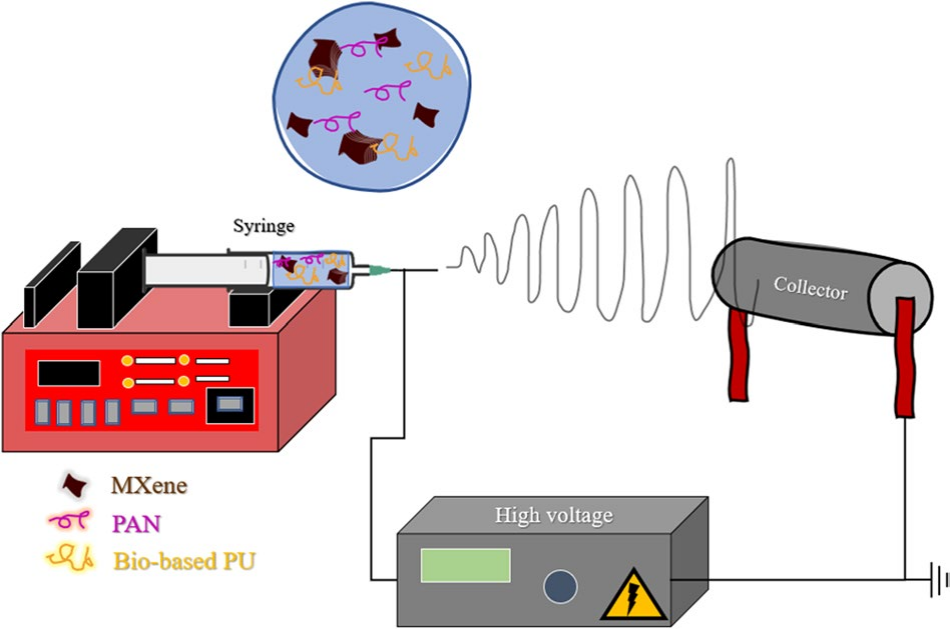
Schematic of the electrospinning process for the preparation of PAN/bio-based PU/Ti_3_C_2_T_x_ MXene electrospun membrane.

PAN/bio-based PU/Ti_3_C_2_T_x_ MXene electrospun membranes with different Ti_3_C_2_ MXene contents in the range of 0–10 wt% were prepared via the electrospinning technique. The equipment consisted of a high-voltage power supply, volumetric syringe pump, and fiber collector, which were supplied by IBA Company, Thailand. All equipment were placed in a chamber, and the humidity and temperature during electrospinning experiment were controlled at 40% ± 3% and 23 °C ± 2 °C, respectively. The PAN/bio-based PU with the weight ratio of 75/25 and 8 wt% concentration was dissolved in DMF solvent overnight until a yellow homogeneous solution was obtained. Ti_3_C_2_T_x_ MXene was dispersed in DMF separately and sonicated for 2 h. The polymer and Ti_3_C_2_ MXene solution were then mixed and further sonicated for 2 h. The dark solution of PAN/bio-based PU/Ti_3_C_2_T_x_ MXene was noticed. Figure S2 depicts the preparation process of PAN/bio-based PU/Ti_3_C_2_T_x_ MXene. The PAN/bio-based PU/Ti_3_C_2_T_x_ MXene was then fabricated into a non-woven membrane via an electrospinning technique. DMF was selected as a solvent because it has a high vapor pressure and a good solvent for the well dispersion of MXene^30^. The homogeneous solution of PAN/bio-based PU/Ti_3_C_2_T_x_ MXene was collected in a 5 ml syringe coupled with a needle having an inner diameter of 0.5 mm and placed in a syringe pump with a controlled flow rate of polymer solution of 2.0 ml/h. The applied voltage and distance from the tip to collector were set at 26.5 kV and 20 cm, respectively. Figure 1 shows the set-up of the electrospinning process for membrane preparation.

### Characterization

Morphologies of the PAN/bio-based PU/Ti_3_C_2_T_x_ MXene electrospun membranes were determined via a scanning electron microscope (SEM) (Hitachi SU-4800) equipped with an energy dispersive X-ray (EDS) spectroscope. The SEM process was accelerated at a voltage of 3.0 kV and an emission current of 10 mA. The surfaces of samples were sputter-coated with gold before measurement. The average fiber diameter and bead-like structure were from 130 measurements and obtained using Image J.

X-ray diffraction (XRD) (Bruker AXS model D8 Advance, Germany) spectra were acquired using a powder diffractometer with Cu Kα radiation at a step size of 0.02° in the range of 5–80°, accelerating voltage of 40 kV, and emission current of 40 mA.

The porosity of the electrospun membrane was measured using n-butanol uptake test. The samples were then cut into 1.9 cm^2^ pieces. The porosity values were calculated using Eq. (1):

1 $$Porosity \left( \% \right) = \frac{{W_{w} - W_{d} }}{{\rho_{d} V_{d} }}$$

where ${W}_{d}$ is the dry weight of the PAN/bio-based PU/Ti_3_C_2_ MXene electrospun membrane, ${W}_{w}$ is the wet weight of the membrane after immersion of the sample in n-butanol for 2 h, ${\rho }_{b}$ is the density of n-butanol, and ${V}_{d}$ is the volume of dry mats. The average value was calculated from three measurements.

The membrane samples were cut into dimensions of 2 × 2 cm^2^. The electrolyte uptake of the membranes was determined using Eq. (2).

2 $$Electrolyte\; uptake \left( \% \right) = \frac{{W_{w} - W_{d} }}{{W_{d} }} \times 100$$

where ${W}_{d}$ and ${W}_{w}$ are the weight of the PAN/bio-based PU/Ti_3_C_2_ MXene membrane before and after immersion of the sample in the liquid electrolyte for 1 h, respectively. The average value was calculated from three measurements.

The wettability of the membrane by an electrolyte was observed with an electrolyte droplet placed on their surfaces and confirmed by an in-house contact-angle analyzer. The electrospun membrane was dried in a vacuum overnight to remove all moisture. Contact-angle measurements were conducted within 5 s by placing one drop of the electrolyte on samples. The final contact angles were obtained as the average of three measurements at room temperature.

A potentiostat/galvanostat (PSTrace4 Palm Sens) was utilized to investigate the electrochemical properties. Measurements were performed using an applied AC potential of 10 mV from 1 MHz to 1 Hz. The Zn/separator/Zn cell was constructed by membrane insertion between the blocking electrodes made of stainless steel. The membrane thickness and active area were approximately 250–350 μm and 2.834 cm^2^, respectively^4^. Transference number measurements were performed using the DC polarization method via chronoamperometry. A polarization voltage of 10 mV was applied across the samples, and the initial maximum current *I*_0_ and steady-state current *I*_s_ were recorded. The ionic conductivity (σ) can be determined through Eq. (3). The average value was from three measurements.

3 $${\text{s}}\left( {{\text{S}}/{\text{cm}}} \right) = \frac{d}{R S}$$

where d represents the thickness of the separator, R stands for bulk resistance, and S is the area of effective contact between the separator and electrode.

Coin cells (CR2032), including Zn/separator/Zn, were brought to study their electrochemical compatibility using the voltage profile by the Neware battery testing system (Shenzhen Neware CT-4008). The charge–discharge cycles of symmetric Zn//Zn cells were rendered at current densities of 0.25, 0.50, 1.25, and 2.50 mA cm^−2^ for 100 h. The test required 25 h to complete one cycle. Then, the voltage response was plotted against time. The full cell with oxygen defect enriched (NH_4_)_2_V_10_O_25_⋅8H_2_O (NVO) was explained in supplementary. Cyclic voltammetry (CV) was performed on a PalmSens4 potentiostat (DutchDisclaimer) with scan speeds of 0.1 mV s^−1^. The battery performance was studied using a LAND (CT2001A) battery tester (Wuhan, China) at different current densities in the 0.50–1.50 V (vs. Zn/Zn2 +) voltage window for Zn//NVO.

The thermal dimension stability of the membrane was determined. The circular shape sample having a diameter of 19 mm was heated in an oven at a temperature of 150 °C for 1 h.

## Results and discussions

### Morphology of PAN/bio-based PU/Ti_3_C_2_T_x_ MXene composite

**Figure 2:**
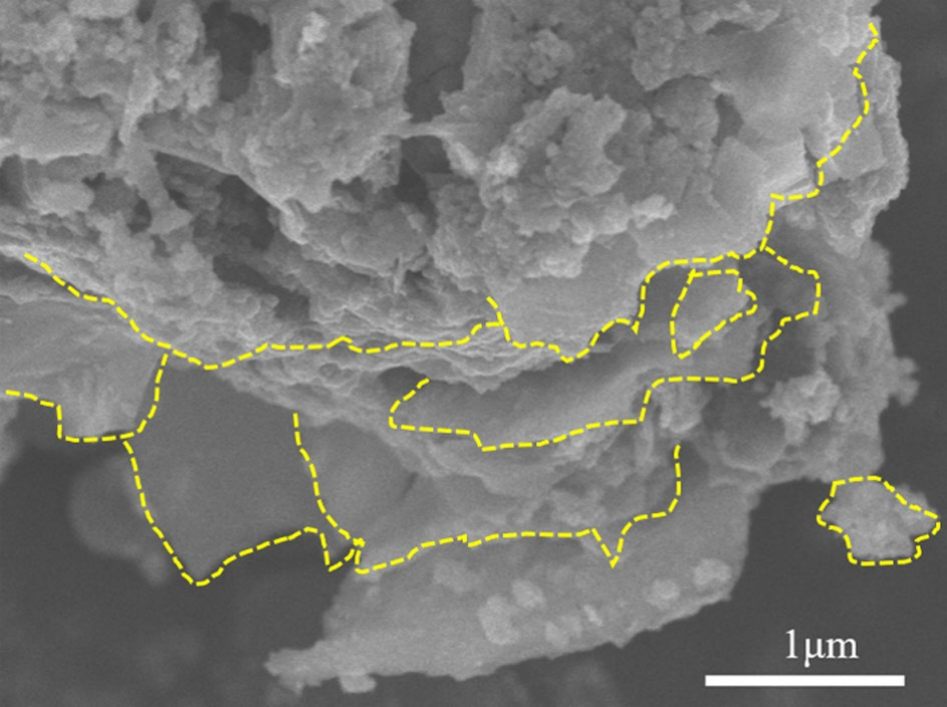
SEM images of Ti_3_C_2_T_x_ MXene micro-nano sheet.

The morphology of pristine Ti_3_C_2_T_x_ MXene microsheet was investigated by SEM (Fig. 2). The multi-layer and well-aligned architecture of Ti_3_C_2_T_x_ MXene microsheet can be observed, and the sheet thickness was approximately 1.5–2 µm. With the etching of Al in the MXene production process, the bulky objects became wrinkled, and stacked sheets with minor gap voids were observed between the interconnected sheets. A few-layer stacked agglomeration may result from the restacking of sheets during the drying process.

**Figure 3:**
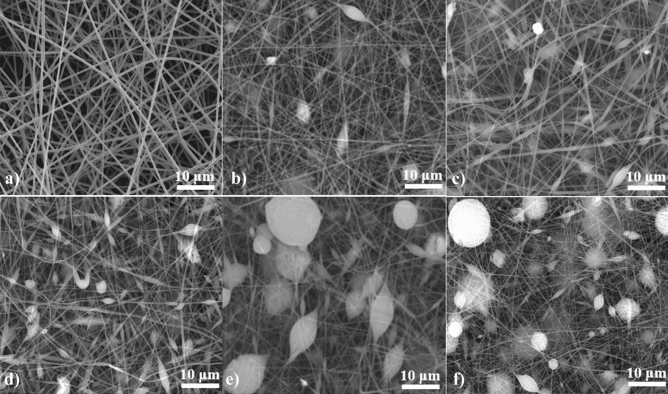
Morphology of PAN/bio-based PU/Ti_3_C_2_T_x_ MXene composite membrane with different Ti_3_C_2_T_x_ MXene contents: (**a**) 0 wt% MXene, (**b**) 0.1 wt% MXene, (**c**) 1 wt% MXene, (**d**) 5 wt% MXene, (**e**) 7 wt% MXene, and (**f**) 10 wt% MXene. Preparation conditions: applied voltage of 26.5 kV, 20 cm distance from the tip to the collector, and solution flow rate of 2 ml/h.

**Figure 4:**
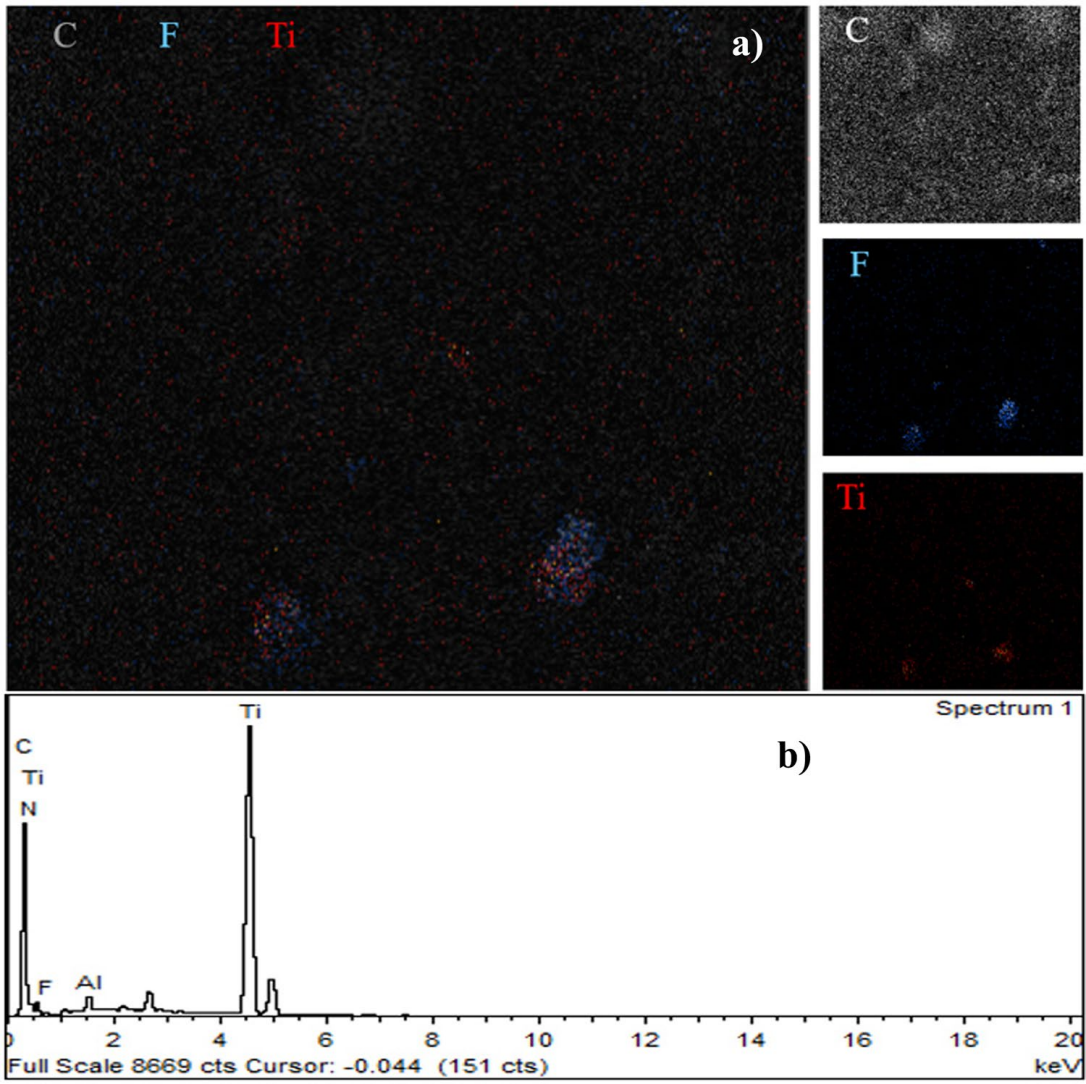
Elemental analysis of PAN/bio-based PU/Ti_3_C_2_T_x_ MXene composite membrane: (**a**) element mapping images and (**b**) EDX spectrum indicating Ti in the nanofiber.

The pure PAN/bio-based PU electrospun fiber was prepared in advance to find suitable processing conditions before the addition of Ti_3_C_2_T_x_ MXene. Figure 3 shows that the PAN/bio-based PU at a concentration of 8 wt% and electrospinning conditions of 26.5 kV applied voltage and 20 cm distance from the tip to collector showed smooth and continuous fibers without the appearance of beads. Therefore, the PAN/bio-based PU/Ti_3_C_2_T_x_ MXene electrospun with 0.1–10 wt% Ti_3_C_2_T_x_ MXene was fabricated using the same conditions. In the presence of Ti_3_C_2_T_x_ MXene, the fiber morphology changed (Fig. 3), and a bead-like structure was observed (magnified in Figure S3). Then, the several micron size fibers of commercial glass microfiber was shown in Figure S4. The backscattered electrons (BSE) of SEM were obtained to confirm the presence of Ti_3_C_2_ MXene in the nanofiber. The BSE images presented high sensitivity to different atomic numbers of elements. The bright spot of a material indicates a high atomic number. Ti in Ti_3_C_2_T_x_ MXene has an atomic number of 22, which is notably higher than those of the backbone of polymer chain containing C, N, and O, which have atomic numbers of 6, 7, and 8, respectively. Therefore, continuous and well-distributed Ti_2_C_3_ MXene in PAN/bio-based PU composite fibers was successfully prepared when up to 10 wt% Ti_3_C_2_T_x_ MXene content was used (Table S1). Elemental mapping was conducted on a fiber of electrospun membrane fabricated from a solution containing 10 wt% Ti_3_C_2_T_x_ MXene (Fig. 4a). From Fig. 4b, the EDS spectrum also showed a peak of Ti from Ti_3_C_2_. The result confirmed the presence of Ti from Ti_3_C_2_T_x_ MXene in the PAN/bio-based PU fiber, and F was probably generated from the etching process during MXene synthesis. The high amount of Ti_3_C_2_T_x_ MXene resulted in an extremely high-viscosity polymer solution and hindmost spinnability for fiber formation. With the incorporation of 0.1–10 wt% Ti_3_C_2_T_x_ MXene, changes in the fiber morphology of the obtained membrane were observed as Ti_3_C_2_T_x_ MXene was embedded in the fiber structure. As presented in Table S1, the diameter significantly decreased from 510 nm in the pure membrane to approximately 220–240 nm in the 0.1–10 wt% Ti_3_C_2_T_x_ MXene composite membrane. This result was due to the polymer chains that elongated to encapsulate the Ti_3_C_2_ MXene microsheet, thus decreasing the fiber size. The size of Ti_3_C_2_T_x_ MXene embedded in the fiber measured 2.1 ± 0.8 µm in the 0.1 wt% sample, and this finding implied a relatively broad size distribution. The size decreased significantly to 2.0 ± 0.6 µm in the sample with 5 wt% Ti_3_C_2_T_x_ MXene. The high amount of Ti_3_C_2_T_x_ MXene resulted in the agglomeration of particles, which measured 5.1 ± 2.8 and 4.2 ± 2.6 µm in the PAN/bio-based PU composite/Ti_3_C_2_ MXene composite fiber with 7 and 10 wt% Ti_3_C_2_T_x_ MXene, respectively. This characteristic was also observed in the system of PVDF/halloysite nanotube^31^ and polyethylene oxide/Ti_3_C_2_^32^. The results showed that the PVDF fiber diameter decreases in the presence of inorganic.

**Figure 5:**
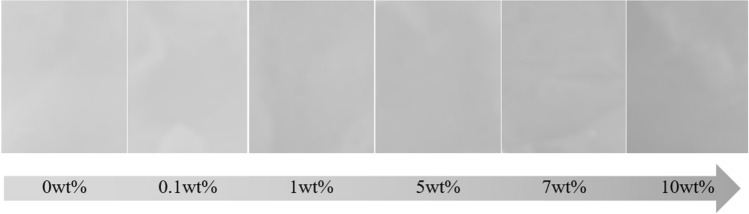
Optical images of PAN/bio-based PU/Ti_3_C_2_T_x_ MXene composite membrane with different MXene contents.

The physical appearance of the Ti_3_C_2_T_x_ MXene composite membrane was further investigated (Fig. 5). The color of the composite gradually changed with Ti_3_C_2_T_x_ MXene concentrations, i.e., from the pearl white pure PAN/bio-based PU membrane to the light-gray Ti_3_C_2_T_x_ MXene composite membrane. This result was due to the increased amount of black metal powder of MXene and well particle distribution. However, the color was different from that of a MXene/PAN nanofiber that showed a dark membrane due to the use of nanosheet MXene^33^.

The mechanical appearance was evidently depicted by the folding test. Figure S5 displays the flexibility of the PAN/bio-based PU/Ti_3_C_2_T_x_ MXene composite membrane, which revealed the high flexibility of the Ti_3_C_2_T_x_ MXene composite membrane without cracking. The fiber/MXene composite showed a robust mechanical flexibility without any other additives, benefiting from the high porosity of the electrospun membrane. However, rigid particles were embedded in the fiber structure. In addition, the highest tensile strength was 1.68 ± 0.14 MPa for the 5% MXene content of PAN/bio-based PU/Ti_3_C_2_T_x_ MXene composite membrane. The result indicated the suitable application of composite membranes in flexible electronic devices.

### XRD results of PAN/bio-based PU/Ti_3_C_2_T_x_ MXene composite

Figure S6 exhibits the XRD pattern of pristine Ti_3_C_2_T_x_ MXene, with the crystalline peaks at the (002), (004), (006), (101), (103), (104), (105), (107), and (108) plane corresponding to 2θ of 9.7°, 18.3°, 23.2°, 34.1°, 36.8°, 38.9°, 41.8°, 50.6°, and 52.4°, respectively. The XRD result corresponded to that reported by Cao Y (2017)^34^. Among these peaks, the (002), (004), (006) (008), and (105) planes were attributed to the MXene structure. The peak intensity at 2θ of 38.9° was related to the Al layer and revealed some MAXphase residue in the Ti_3_C_2_T_x_ MXene^35^. The XRD result corresponded to the EDS finding, which showed negligible traces of Al compared with the Ti and C peaks^36^.

**Figure 6:**
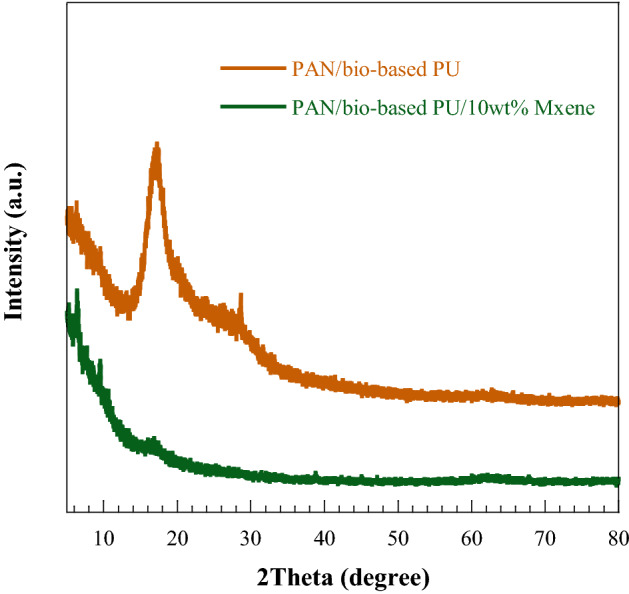
XRD patterns of (**a**) pure PAN/bio-based U and (**b**) PAN/bio-based PU with 10 wt% Ti_3_C_2_T_x_ MXene.

The XRD patterns of the pure PAN/bio-based PU and its Ti_3_C_2_T_x_ MXene composites were determined (Fig. 6). The neat polymer exhibited the ordered packing of the polymer backbone with a crystalline region at 2θ = 17° and a broad peak near 26°, indicating the amorphous region of the nanofiber. With the incorporation of Ti_3_C_2_T_x_ MXene, the peak intensity was broadened, showing the reduced crystalline structure in the polymer via the intercalated structure of the composite polymer chain^16^. The decreased crystalline or increased amorphous regions of the polymer matrix enhanced the electrolyte uptakes and ionic conductivities of the membrane^37^.

### Fourier transform infrared spectroscopy (FTIR) spectra of PAN/bio-based PU/Ti_3_C_2_T_x_ MXene composite membrane

**Figure 7:**
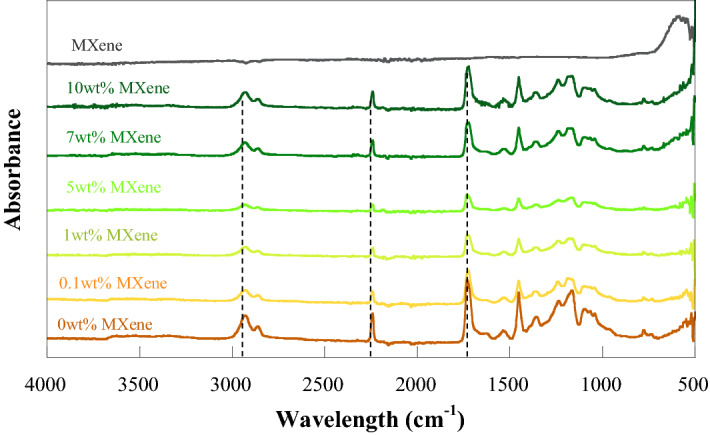
FTIR spectra of PAN/bio-based PU/Ti_3_C_2_T_x_ MXene composite membranes with different Ti_3_C_2_T_x_ MXene contents.

The chemical interaction of PAN/bio-based PU/Ti_3_C_2_T_x_ MXene composite with Ti_3_C_2_T_x_ MXene contents was studied by the FTIR technique. From Fig. 7, the characteristic peaks of the urethane linkage were observed, i.e., the broad absorption peaks at 3434 and 1536 cm^−1^ were related to N–H stretching and bending vibrations of the urethane linkage, respectively, whereas the peak the wavenumber of 1724 cm^−1^ was assigned to C=O stretching^38^. Moreover, the FTIR spectra at 2930 and 1450 cm^−1^ were attributed to the asymmetric stretching and bending vibrations of the methylene group of PAN, respectively. In addition, the absorption peak at 2242 cm^−1^ was assigned to the CN group of PAN^39^. MXene exhibited a characteristic peak at 550 (500–650) cm^−1^, and it was assigned to the Ti–O tensile vibration, whereas the presence of the functional groups –O and –OH of MXene was confirmed from the peak of C–O stretching at 920–1160 cm^−1^ and O–H bending vibrations at 1400–1650 cm^40^, corresponding to the results of a previous report^40,41^. With the addition of Ti_3_C_2_ MXene, no characteristic peak of PAN/bio-based PU was changed, indicating that no chemical interaction occurred between Ti_3_C_2_T_x_ MXene and the polymer matrix. This behavior was also found in the system of PU/nanoclay^42^.

### Porosity and electrolyte uptake of PAN/bio-based PU/Ti_3_C_2_T_x_ MXene composite membrane

**Figure 8:**
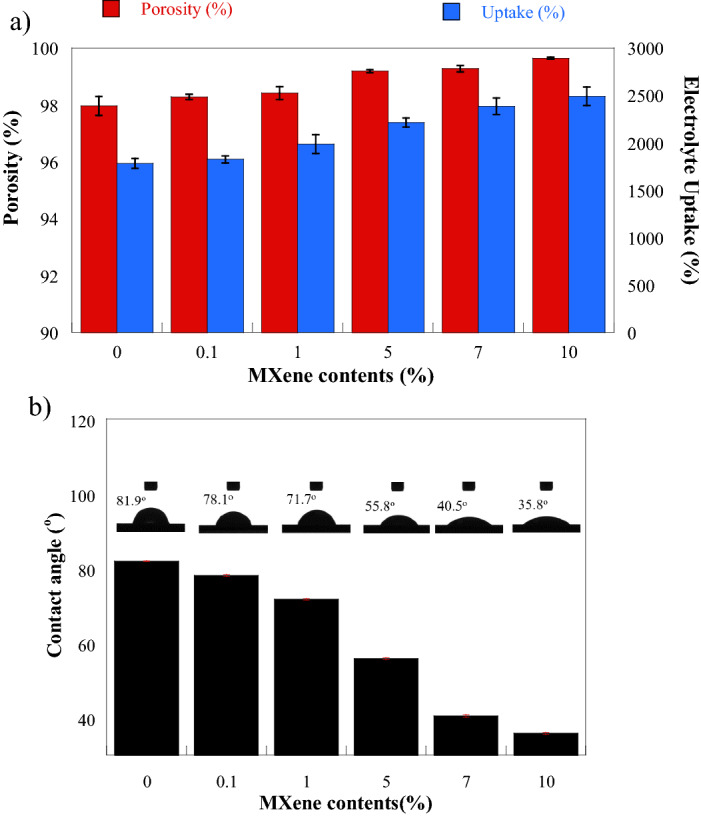
Representative (**a**) porosity (red) and electrolyte uptake (blue) and (**b**) schematic of contact angle test of PAN/bio-based PU/Ti_3_C_2_T_x_ MXene composite.

As electrolyte reservoir and ionic transfer media of separator, the porosity of the sample is an important parameter determining the separator performances. The porosity values of PAN/bio-based PU electrospun membrane and their Ti_3_C_2_ MXene composites were in the range of 97.97–99.65% (Fig. 8a). The porosity was consistent with the increase in MXene content up to 99.65%. The main reason is that the nanofiber membranes had a random 3D network structure, and the fibers overlapped with each other to form the pore structure. According to the morphology in Fig. 3, all MXene composite fibers showed smaller diameters compared with the neat polymer fiber. Moreover, they presented some spindle-like fiber structures and complex pore structures, which probably caused the increase in the porosity value. The porosity value obtained from this work was greater than that reported for PVDF/cellulose acetate/AgTiO_2_ composite system (88%)^43^. The increase in porosity with the increase in inorganic filler was revealed in the separator prepared from the halloysite nanotube PVDF-cellulose acetate^31^.

The wettability of the sample composite electrospun fibers was evaluated by water contact-angle measurement (Fig. 8b). The results showed that the contact angle decreased with the increase in MXene contents in the composite membrane. The flatness of droplets on the sample denoted the development of wettability of the sample. This improvement trend was similar to that reported by Awasthi et al. The improved wettability of the membrane with the increased amount of MXene can be related to the hydrophilicity of MXene due to the presence of hydroxyl or oxygen moieties as terminal groups^44^. The high wettability of the separator membrane indicates its good capability to absorb electrolyte solutions. Moreover, it supports low internal ionic resistance in batteries and extends electrolyte retention in battery cells^1^.

Electrolyte uptake, that is, the liquid electrolyte absorptivity of the separator, is a crucial property. Liu et al. reported an electrolyte uptake of 240% in the nanosilica coated on a conventional PP separator^45^. Liang et al. also developed an electrospinning fibrous separator from PVDF, and it presented more than 400% electrolyte uptake^3^. Large liquid electrolyte uptake is essential for separators because the amount of liquid electrolyte between electrodes can affect the internal ionic resistance of batteries. The electrolyte uptake of PAN/bio-based PU/Ti_3_C_2_T_x_ MXene composite was in the range of 1,783% to 2,491% as plotted in Fig. 8a. The value increased with Ti_3_C_2_T_x_ MXene content and reached a maximum value at 10 wt% Ti_3_C_2_T_x_ MXene. The relationship between electrolyte uptake and porosity showed similar trends. The increase in electrolyte uptake was attributed to the high compatibility of the separator to the liquid electrolyte.

### Ionic conductivity and interfacial resistance of PAN/bio-based PU/Ti_3_C_2_T_x_ MXene composite

Separators, which are porous structure materials, normally provide an ion transport channel, which is related to the ionic conductivity of batteries. AC impedance spectroscopy was used for the investigation. The ionic conductivity was calculated using Eq. (3), in which the intercept of the Nyquist plot at high frequency was used on the real Z as the bulk resistance (Rb). Figure S7 shows the Nyquist plot of PAN/bio-based PU/Ti_3_C_2_T_x_ MXene composite, and Figure S7 (inset) presents the ionic conductivity calculated from the interceptional point on x axis. The results showed that ionic conductivities of Ti_3_C_2_T_x_ MXene composite were all greater than that of the pure PAN/bio-based PU fiber. Moreover, the ionic conductivity increased with the increase in Ti_3_C_2_T_x_ MXene compositions. According to porosity and electrolyte uptake, all MXene addition caused the increase in porosity and electrolyte uptake, which denoted the increase in carrier channels in the same volume of separator and the improvement of zinc ion mobility. Moreover, it was attributed to extensive ion-accessible surface of Mxene and surface functional groups which was reported to play a positive role in reorganizing the structure of polymer matrix chain to concentrate free ion^46–48^.

Table 1 shows the ionic conductivities observed in this work. The highest ionic conductivity was up to 3.35 mS cm^−1^ from 5% Ti_3_C_2_T_x_ MXene content. The ionic transport capability of PAN/bio-based PU electrospun fiber increased with the increase in Ti_3_C_2_T_x_ MXene contents. However, the ionic conductivity decreased at high loading of Ti_3_C_2_T_x_ MXene. This phenomenon was probably due to the tortuous ion transport channel and high crystallinity of the separator associated with high filler contents. The turnover tendency of ionic conductivity was similar to that reported for MXene-modified poly (ethylene oxide) and PVDF–hexafluoro propylene ^12,13^. Table 2 summarizes the performances of composite separators. The ion transference number was calculated using the currents at the steady and initial states in chronoamperometry (Figure S8). The Zn^+^ transference numbers were 0.49 and 0.54 for the glass microfiber and PAN/bio-based PU/5 wt% MXene membranes, respectively. The high ion transference number was due to the porous structure of PAN/bio-based PU/5 wt% MXene membrane.

**Table 1:** Ionic conductivity of PAN/bio-based PU/Ti_3_C_2_T_x_ MXene composite.

Mxene content (%)	Ionic conductivity (mS cm^−1^)
0	1.10 ± 0.24
0.1	1.32 ± 0.28
1	2.71 ± 0.84
5	3.35 ± 0.43
7	2.91 ± 0.51
10	2.36 ± 0.23

**Table 2:** Review of electrospun composite separator for metal ion batteries.

System	Electrolyte uptake (%)	Tensile strength (MPa)	Ionic conductivity (mS cm^−1^)	Refs.
PAN/bio-based PU/Ti_3_C_2_T_x_ MXenes	2214 ± 49	1.68	3.35	Present study
MOF/PAN	794 ± 30 860 ± 31	–	2.83 1.62	^49^
Graphene oxide/PU	733.3	3	3.73	^50^
Halloysite nanotube/CA/PVdF	311	7.6	1.36	^31^

### Electrochemical compatibility of PAN/bio-based PU/Ti_3_C_2_T_x_ MXene composite

**Figure 9:**
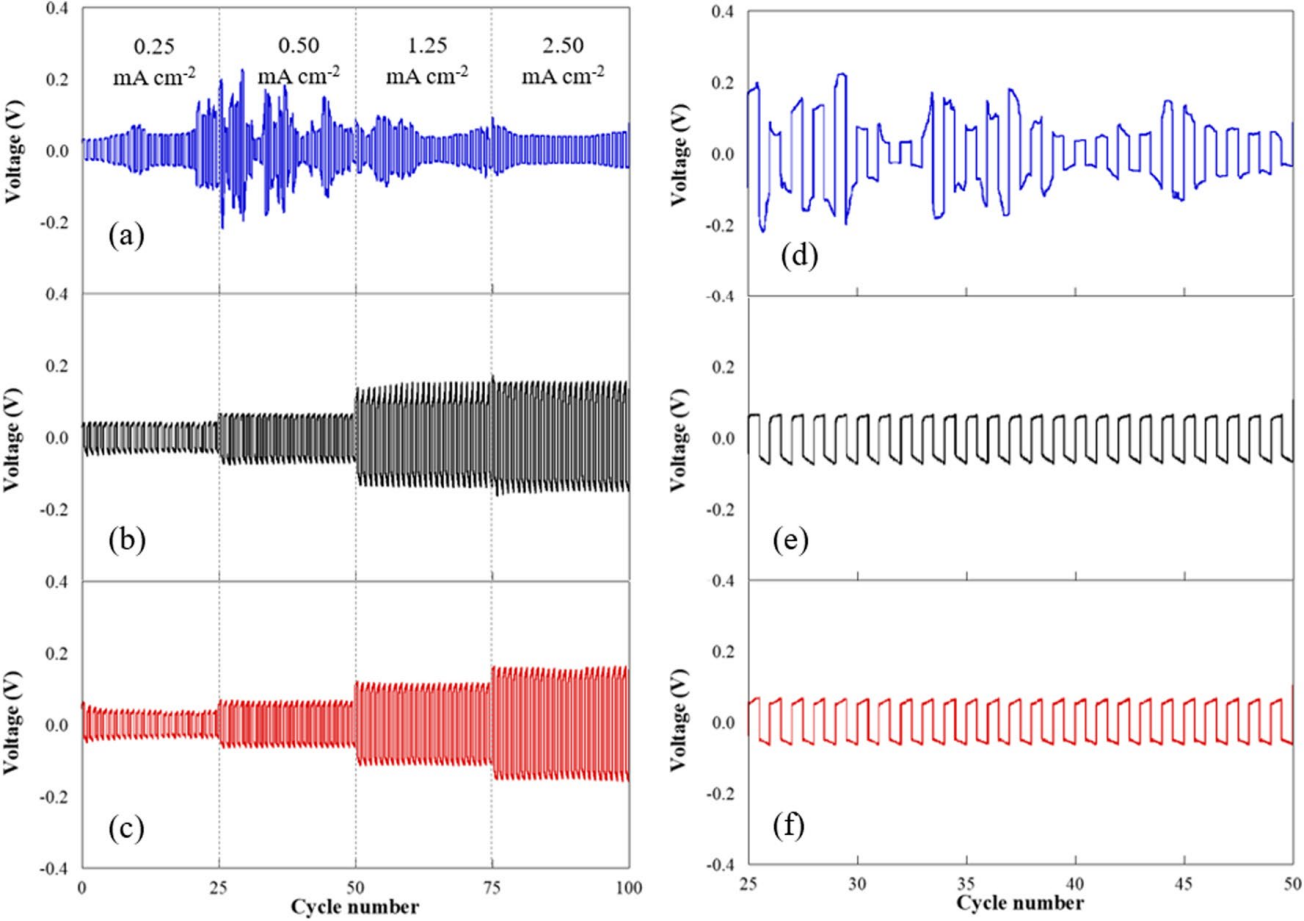
Zn symmetry cell testing for charge/discharges cycle of (**a**) glass fiber separator, (**b**) PAN/bio-based PU without MXene, and (**c**) PAN/bio-based PU/5 wt% MXene; voltage profile in 25 to 50 cycles of (**d**) glass fiber separator, (**e**) PAN/bio-based PU without MXene, and (**f**) PAN/bio-based PU/5 wt% MXene.

Figure 9 shows the typical cycling performance and the voltage profile over long-term charge/discharge cycles at room temperature. The polarization voltage was plotted versus the operating time. The assembly of separators in Zn symmetrical batteries was tested with current density from 0.25 to 2.50 mA cm^−2^. From Fig. 9a, the plot of glass microfiber separator presented a non-stable voltage signal in each current density and caused a short circuit during the cycle process, which revealed the penetration of zinc dendrite on the membrane^51^. The electrospun membrane separator from PAN/PU and its composite with MXene (Fig. 9b and c) displayed stable voltage profiles and presented no short circuit signal. The comparison of voltage stability is presented in the zoom-in charge/discharge process in Fig. 9d–f. In addition, PAN/bio-based PU/5 wt% MXene presented a slightly lower voltage overpotential than neat PAN/bio-based PU, which reflected the lower internal resistance of the symmetric Zn/(PAN/bio-based PU/5 wt% MXene)/Zn cell. The excellent overpotential was due to the good compatibility of materials between the membrane and electrode and sufficient electrolyte in the membrane^52^ and surface functional groups which was reported to play a positive role in reorganizing the structure of polymer matrix chain to concentrate free ion^47,48^. The non-short circuit and stable voltage profile of PAN/bio-based PU/5 wt% MXene has shown great potential for use in safe Zn battery applications. In addition, the zinc electrode after charge/discharge testing was investigated and the morphology observed by SEM are shown in Figure S9. From Figure S9(a), relative smooth surface was noticed from PAN/bio-based PU/5 wt% MXene indicating the uniform zinc electrodeposition inducing by halogenated side of MXene. Whereas glass microfiber separator (Figure S9(b)) showed great bunch of flakes like structure representing the formation of zinc dendrite on the electrode.

**Figure 10:**
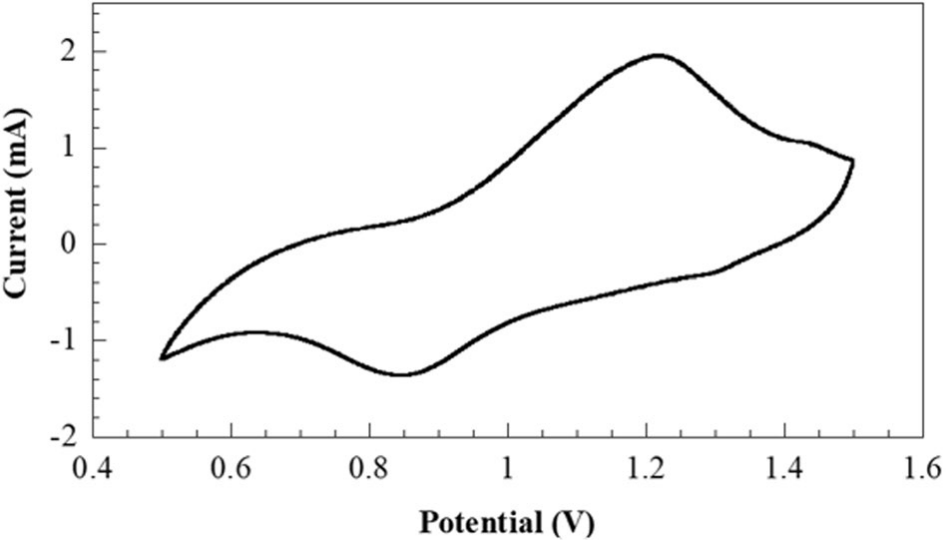
Curve present cyclic voltammetry profile at scan rate 1 mV s^−1^ of full cell NVO//Zn battery.

The full cell assembly was performed to confirm the performance of PAN/bio-based PU/5 wt% MXene as a separator in battery applications. The full cell with vanadium-based cathode and zinc anode (NVO//Zn) was prepared. The cycling voltammetry (CV) of the battery having PAN/bio-based PU/5 wt% MXene as separator is shown in Fig. 10. The first oxidation peak was observed at oxidation potential of 1.21 V whereas the reduction potential of 0.85 V represented as the reversible peak. The complete loop of oxidation and reduction in CV curve was an evident to confirm the usable battery cell. Besides, the rate performance plot was obtained from LAND battery tester. The NVO//Zn battery with PAN/bio-based PU/5 wt% MXene presented the functionable battery cell from various rate as shown in Figure S10. The specific capacity decreased with increasing current rate due to aggravated polarization^53^.

### Thermal stability of PAN/bio-based PU/Ti_3_C_2_ MXene composite

**Figure 11:**
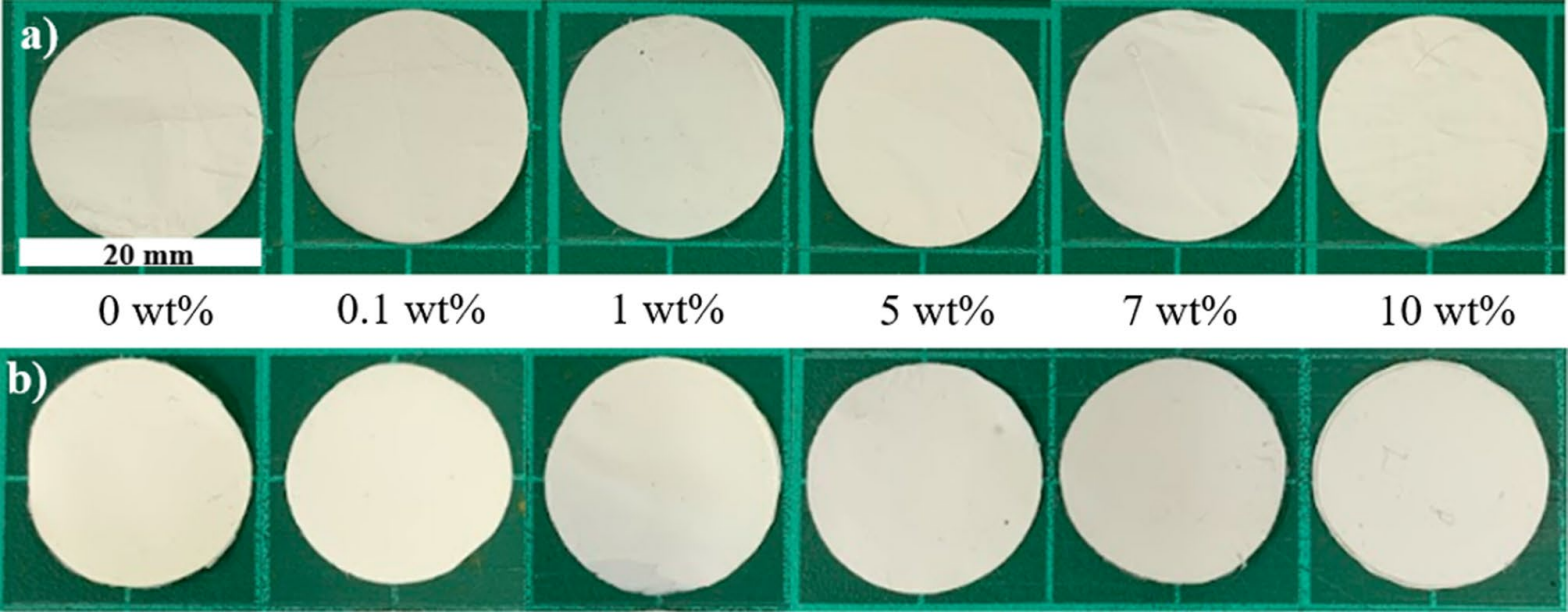
PAN/bio-based PU/Ti_3_C_2_T_x_ MXene composite (**a**) before and (**b**) after heating at 150 °C for 1 h.

In the practical use of a separator, the battery cell may generate some temperature during the operation. As conventional thermoplastic materials restrict the thermal stabilities of battery separators, dimensional change or shrinkage of the samples may result in an electrical short circuit or thermal runaway, which is particularly problematic^54^. In this work, thermal stabilities of the PAN/bio-based PU/Ti_3_C_2_T_x_ MXene composite were observed after continued heating at a high temperature of 150 °C for 1 h. Figure 11 presents the appearances and morphologies of the membranes before and after heating. The appearances of all samples after heating indicated that the membranes can retain their shape without dimensional changes. Moreover, the SEM micrograph of the membranes after heating (Figure S11) showed that the high porous structure of the membrane was retained with some swollen fiber. Moreover, no close pores were observed. After heating, all samples presented porous structures and open pores. By comparison, the large fiber diameter and increased pore structure under the high MXene content were observed after heating. The result indicated that with the incorporation of Ti_3_C_2_T_x_ MXene, the thermal dimensional stability of PAN/bio-based PU can be enhanced. However, under severe heating conditions of 150 °C and 180 °C for each 1 h, evident dimensional changes were observed (Figure S12). The samples shrank by approximately 2–3 mm due to the high temperature and long treatment time.

**Figure 12:**
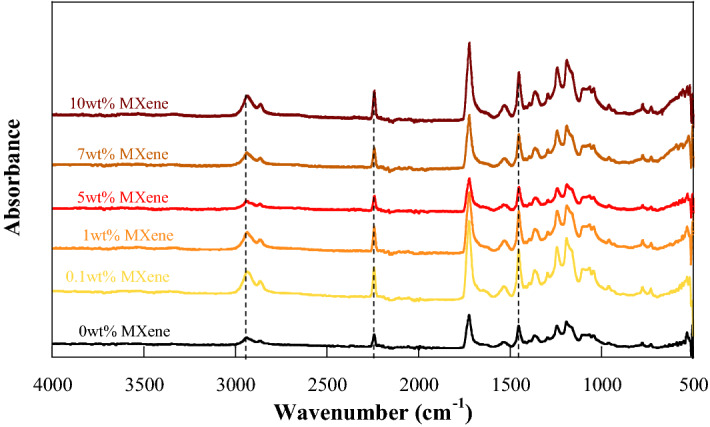
FTIR spectra of PAN/bio-based PU/Ti_3_C_2_T_x_ MXene composite membrane with different Ti_3_C_2_ MXene contents after heating at 150 °C for 1 h.

In addition, the FTIR spectra of PAN/bio-based PU/Ti_3_C_2_T_x_ MXene composite membrane after heating were determined (Fig. 12). The FTIR spectra of PAN/bio-based PU after heating illustrated that the absorption bands at 2242 cm^−1^ decreased compared with those of the sample before heating, indicating the cyclization reaction between adjacent –CN in PAN structure. In the same manner, the absorption peaks at 2930 and 1450 cm^−1^, which corresponded to the stretching and bending vibrations of the methyl group, respectively, decreased, revealing the dehydrogenation of the PAN chain^55^. During heat treatment, a weak shoulder appeared at 2211 cm^−1^, and it was due to the conjugation of –CN with the adjacent –C=CH– resulting from a dehydrogenation reaction. A similar behavior was also reported^56^. No significant change in the absorption peak intensity was observed in the PAN/bio-based PU/Ti_3_C_2_T_x_ MXene composite, indicating the thermal barrier effect of the embedded MXene metal phase.

## Conclusion

In summary, PAN/bio-based PU/Ti_3_C_2_T_x_ MXene composite membranes were successfully fabricated by an electrospinning technique. The mixture of polymer solution of PAN and bio-based PU at a ratio of 75/25 exhibited a smooth and continuous electrospun fiber with no formation of beads. Therefore, this polymer ratio was further used to prepare MXene composite nanofibers. MXene was added to the polymer in the range of 0–10 wt%. The SEM micrograph showed that the Ti_3_C_2_ MXene microsheet was embedded in the PAN/bio-based PU fiber, appearing as spindle-like fiber structure. The great spindle-like structure in the nanofiber was observed in the sample with a high Ti_3_C_2_T_x_ MXene content. However, at higher amounts of Ti_3_C_2_T_x_ MXene (5 wt%), a less spindle like structure was noticed, and the size increased, indicating the agglomeration of Ti_3_C_2_T_x_ MXene. The EDX result also confirmed the integration of MXene into the fiber. The composite membrane showed a high flexibility as the mats can wrap around a glass rod without cracking appearance. The integration of MXene in PAN/bio-based PU resulted in an intercalated structure, as noticed from the XRD results. The porosity of electrospun fiber increased with the increase in Ti_3_C_2_T_x_ MXene contents due to the more connecting structure of Ti_3_C_2_T_x_ MXene in the specimen. The outstanding porosity also affected the electrolyte uptake. The ionic conductivity of the sample increased with increased Ti_3_C_2_T_x_ and MXene, resulting from the high porosity and electrolyte uptake. With high electrolyte uptake and ionic conductivity, the stable charge–discharge performance was also presented from PAN/bio-based PU/Ti_3_C_2_T_x_ MXene, and no short circuit signal was detected through cycle testing with varying current densities. However, an extremely high electrolyte uptake caused a reduction in ionic conductivity. The morphology of fiber structure of the composite membrane displayed very little change as the fiber was swollen after heating condition. Moreover, the Ti_3_C_2_T_x_ MXene hindered the decomposition of the polymer composite, as evidenced by the FTIR spectra. Accordingly, the PAN/PU/ Ti_3_C_2_T_x_ MXene composite membrane is a promising separator for applications of high-performance Zn-ion batteries.

## Supplementary Information

Supplementary Information.

## Data Availability

The datasets used and/or analysed during the current study available from the corresponding author on reasonable request.
