# Supplementary material for: High electrolyte uptake of MXene integrated membrane separators for Zn-ion batteries
Source: Sci Rep. 2022 Nov 19;12:19915. doi: 10.1038/s41598-022-24578-8 (PMC9675853; doi:10.1038/s41598-022-24578-8)
Supplement: Supplementary file 1 — Supplementary Information. [file 41598_2022_24578_MOESM1_ESM.docx]

**High electrolyte uptake of MXene integrated membrane separators for Zn-ion batteries**

Chutiwat Likitaporn^1^, Manunya Okhawilai^2,3*^Pornnapa Kasemsiri^4^, Jiaqian Qin^2,3^, Pranut Potiyaraj^3,5^, Hiroshi Uyama^6^

^1^ Nanoscience and Technology Interdisciplinary Program, Graduate School, Chulalongkorn University, Bangkok 10330, Thailand.

^2^ Metallurgy and Materials Science Research Institute, Chulalongkorn University, Bangkok, 10330, Thailand.

^3^ Center of Excellence in Responsive Wearable Materials, Chulalongkorn University, Bangkok, 10330, Thailand.

^4^ Sustainable Infrastructure Research and Development Center and Department of Chemical Engineering, Faculty of Engineering, Khon Kaen University, Khon Kaen, 40002, Thailand.

^5^ Petrochemistry and Polymer Science, Faculty of Science, Chulalongkorn University, Bangkok, 10330, Thailand.

^6^ Department of Applied Chemistry, Graduate School of Engineering, Osaka University, Osaka 565-0871, Japan.

* Corresponding Author E-mail address: [Manunya.o@chula.ac.th](mailto:Manunya.o@chula.ac.th)

**Preparation of cathode**

**1. Materials**

NH_4_VO_3_ (purity > 99.0%), thiourea (purity > 99.0%) were purchased from Carlo Erba Co. Ltd., poly-(vinylidene fluoride) (PVDF; HSV 900) was obtained from Kynar. All of the reagents were of analytical purity and used as received.

**2. Method**

The (NH_4_)_2_V_10_O_25_⋅8H_2_O (NVO) was prepared by following Cao et al. [1]. Commercial NH_4_VO_3_ of 0.468 g (4 mmol) powder was dissolved in 50 mL deionized water at 70 °C in 50 mL deionized water. Then, 0.228g of thiourea (3 mmol) was added into the solution with stirring after the reactants were completely dissolved. Afterwards, the solution was adjusted for pH using diluted sulfuric acid until pH reached 2, then stirred for 30 minutes. Next, the mixture was maintained at 90 °C for another 2.5 h in an oil bath, a dark green solution was obtained. After cooling down to room temperature naturally, the products were collected and washed with deionized water and ethanol, and the final (NH_4_)_2_V_10_O_25_•8H_2_O nanosheets with rich oxygen defects were obtained after drying at 60°C for 24 h in vacuum. Then, the NVO cathode prepared by mixing the as-prepared NVO, conductive carbon and PVDF with the mass ratio of 7:2:1, and then coating the slurry on graphite paper.

a)


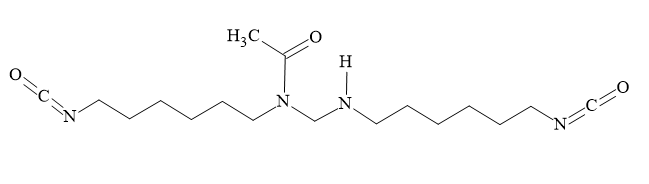


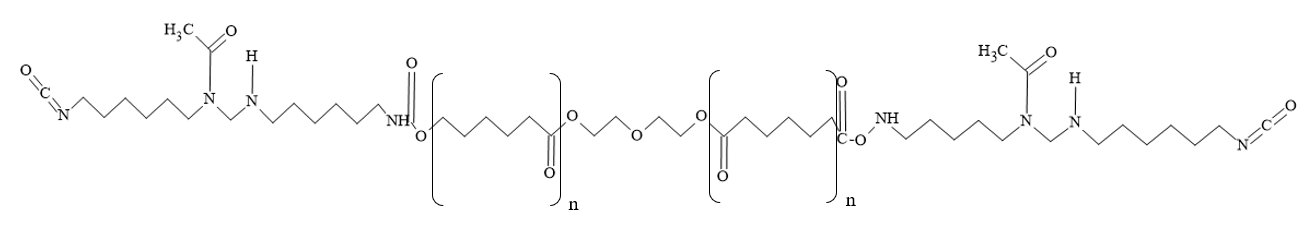


b)

**Figure S1.** Chemical structures of a) bio-based isocyanate and b) bio-based PU.


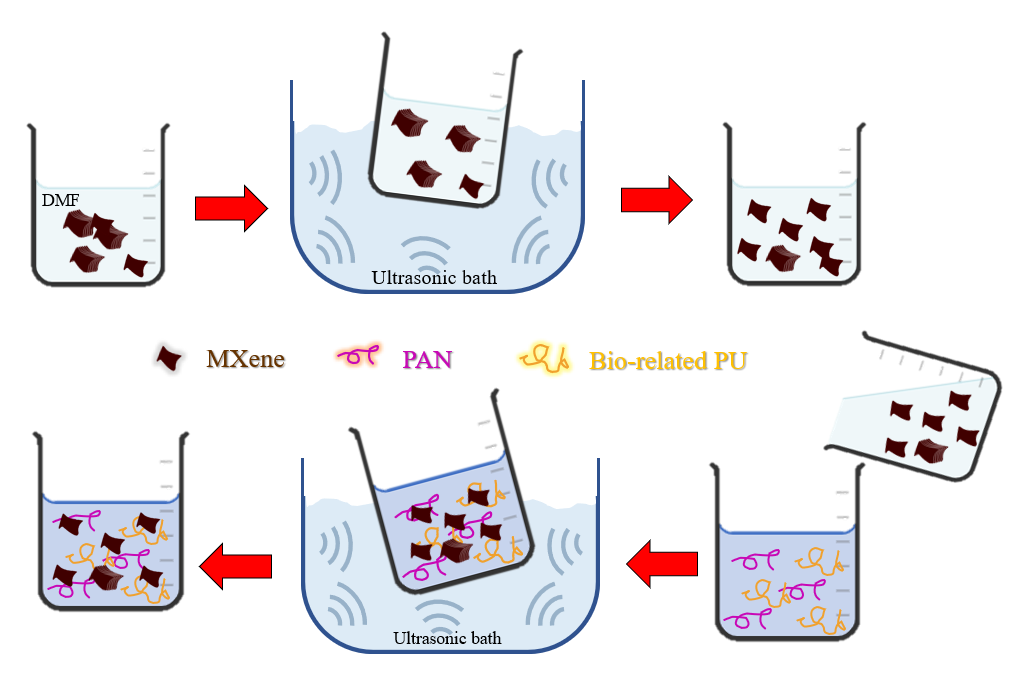


**Figure S2.** Schematic of preparation process of PAN/bio-based PU/Ti_3_C_2_T_x_ MXene solution.


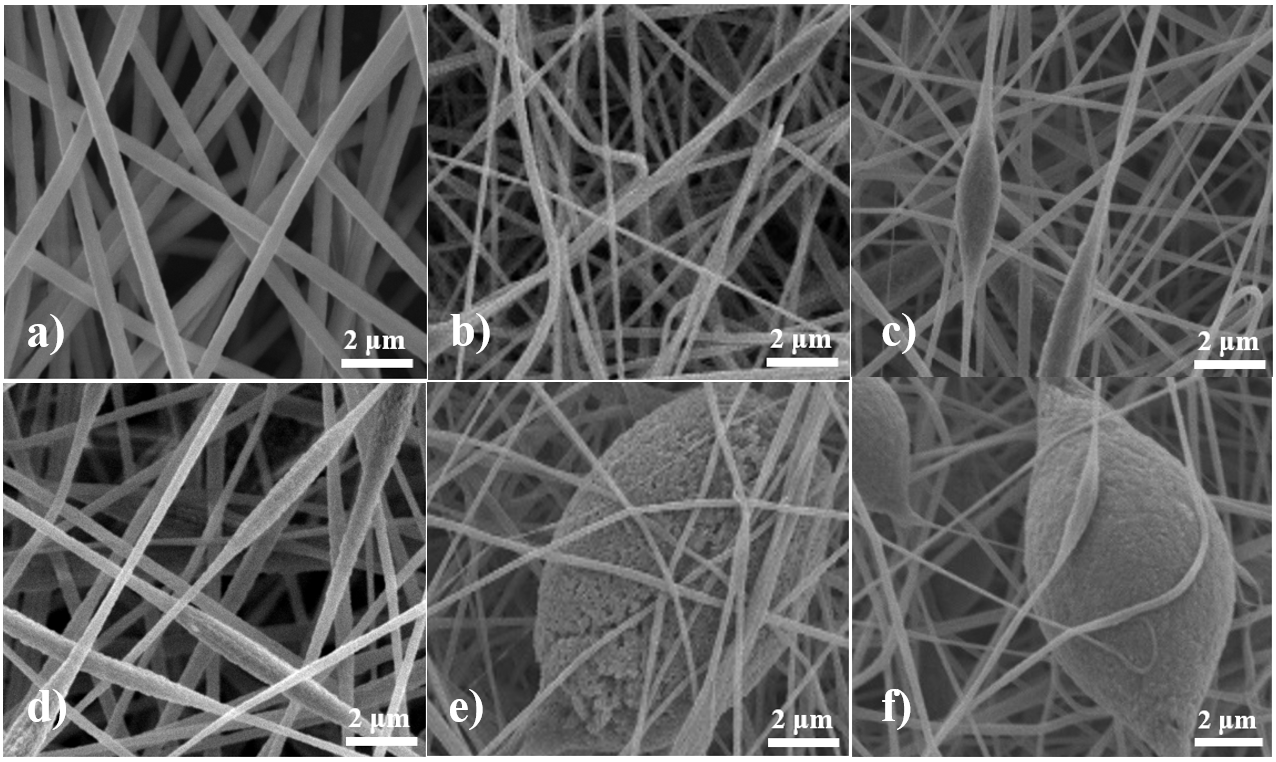


**Figure S3.** Morphology at 5,000 magnifications of PAN/bio-based PU/Ti_3_C_2_T_x_ MXene composite membrane with different Ti_3_C_2_T_x_ MXene contents: a) 0wt% MXene, b) 0.1 wt% MXene, c) 1 wt% MXene, d) 5 wt% MXene, e) 7 wt% MXene and f) 10 wt% MXene at condition for preparation: applied voltage of 26.5 kV, distance from tip to collector of 20 cm and solution flow rate of 2 ml/h.


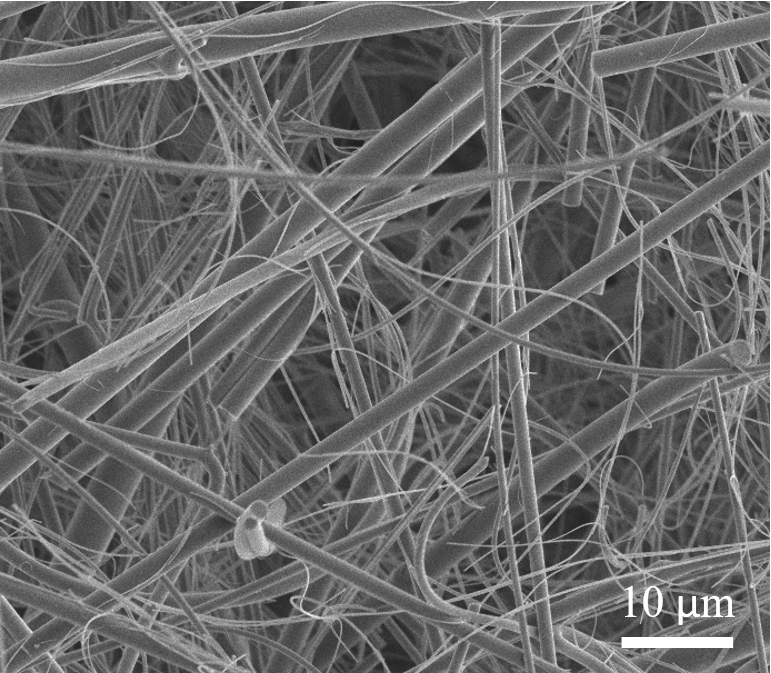


**Figure S4**. SEM image of morphology of glass microfiber filter membrane.


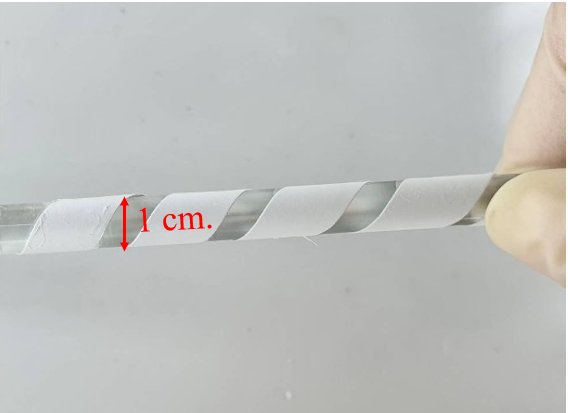


**Figure S5.** PAN/bio-based PU/Ti_3_C_2_T_x_ MXene composite membrane wrapped around glass rod with diameter of 1 cm.

(108)

(006)

(004)

(002)

**Figure S6.** XRD pattern of Ti_3_C_2_T_x_ MXene microsheet.


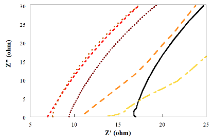

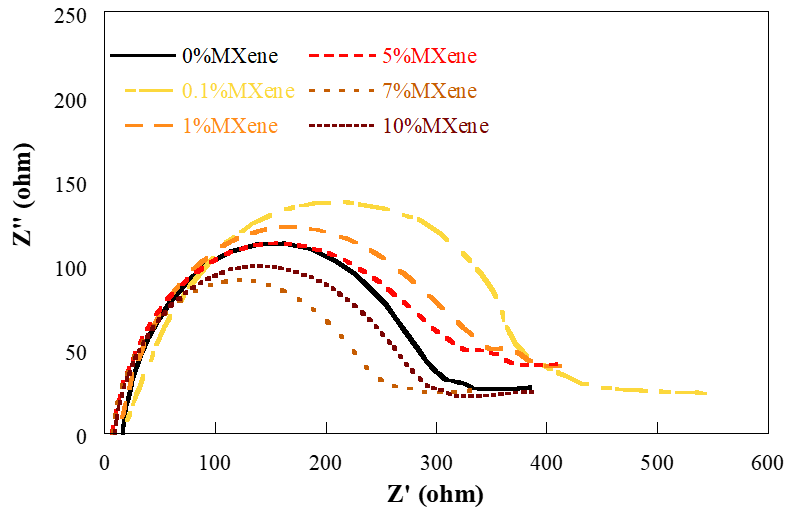


**Figure S7.** Nyquist plot of PAN/bio-based PU/Ti_3_C_2_T_x_ MXene composite and ionic conductivity (inset).


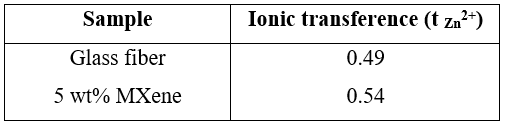

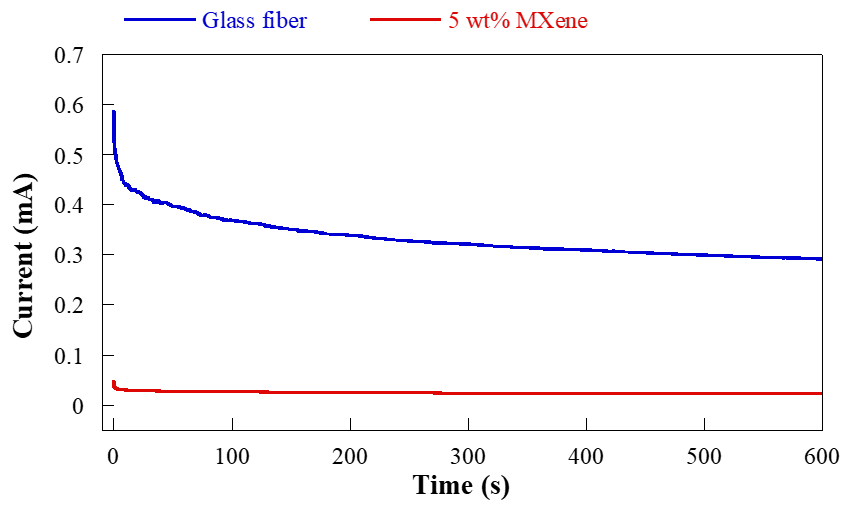


**Figure S8** Representative chronoamperometry profiles at room temperature in block cells using Zn metal as both electrodes with a step potential of 10 mV.


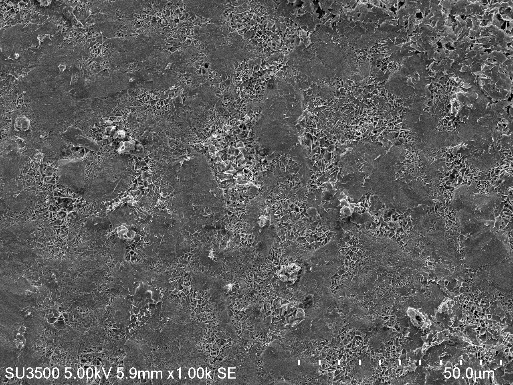

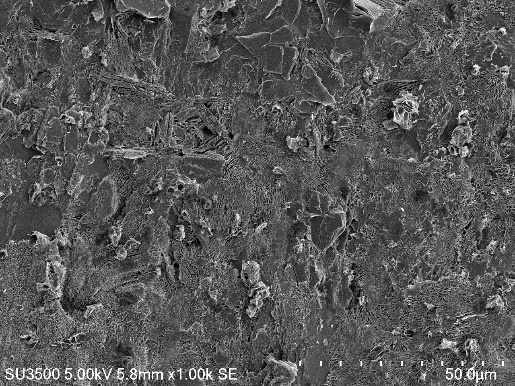


**b)**

**a)**

**Figure S9** Morphology of Zinc electrode after testing of charge/discharge cycle from a) 5 wt% MXene and b) glass microfiber.


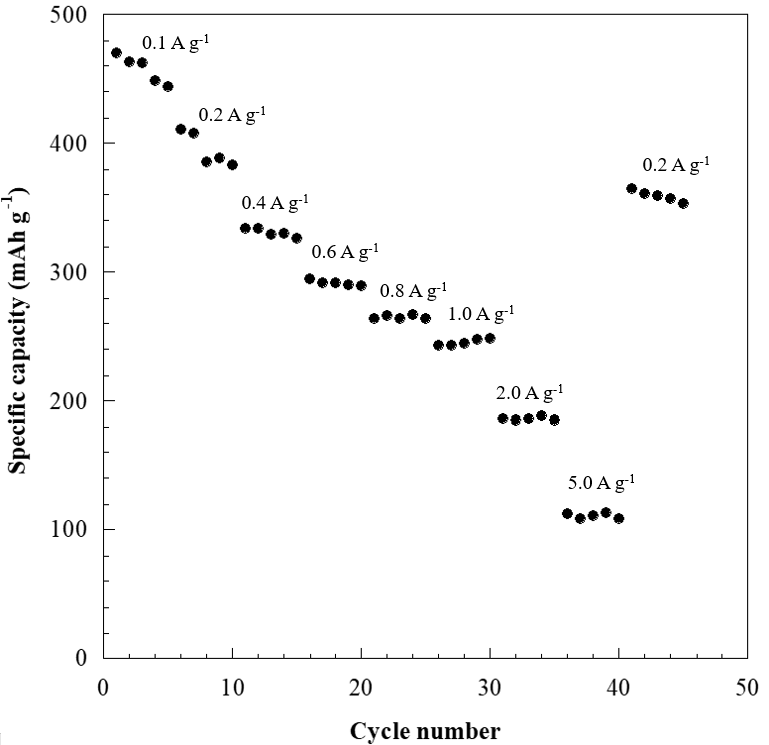


**Figure S10** Rate performance of full cell NVO//Zn battery.


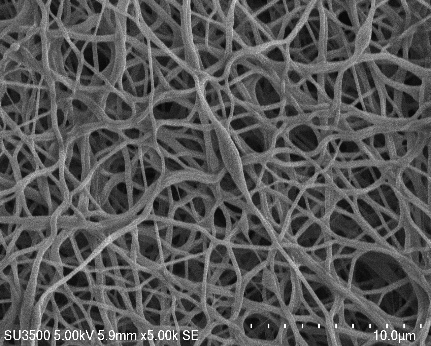


**e)**


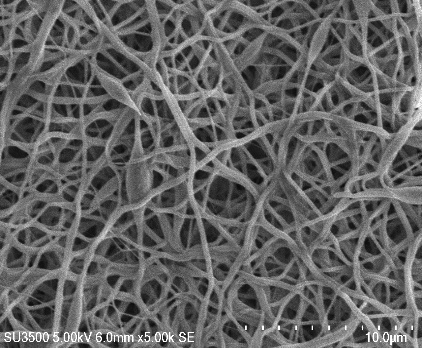


**f)**


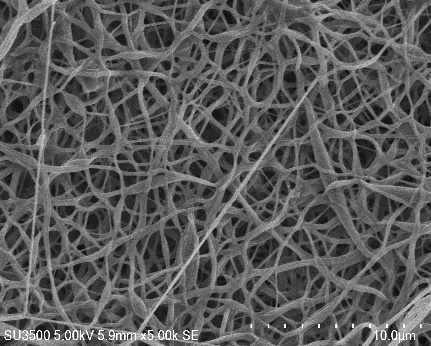


**d)**


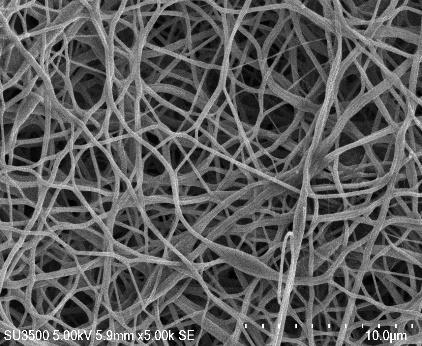


**c)**


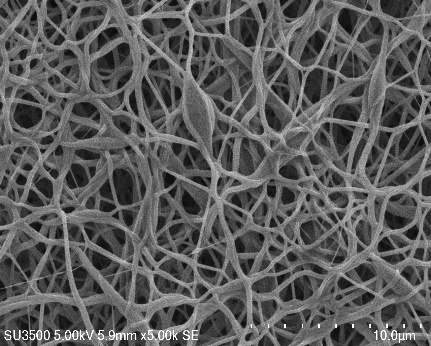


**b)**


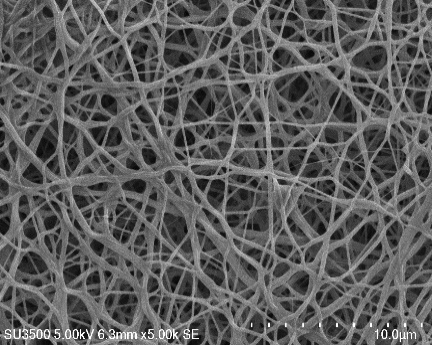


**a)**

**Figure S11.** Morphology of PAN/bio-based PU/Ti_3_C_2_T_x_ MXene composite membrane membrane with different Ti_3_C_2_ MXene contents: a) 0wt% MXene, b) 0.1 wt% MXene, c) 1 wt% MXene, d) 5 wt% MXene, e) 7 wt% MXene and f) 10 wt% MXene after heated at 150°C for 1h.


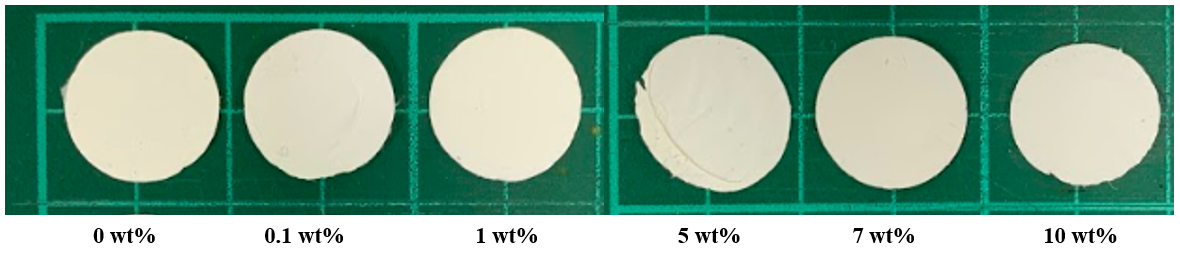


**Figure S12.** Appearance of PAN/bio-based PU/Ti_3_C_2_T_x_ MXene composite after heating at 150°C for 1h and 180°C for 1h**.**

**Table S1** Fiber diameter and MXene in fiber diameter of PAN/bio-based PU/MXene.

| **Sample** | **Fiber diameter** | **MXene in fiber diameter** |
| --- | --- | --- |
| PAN/bio-based PU | 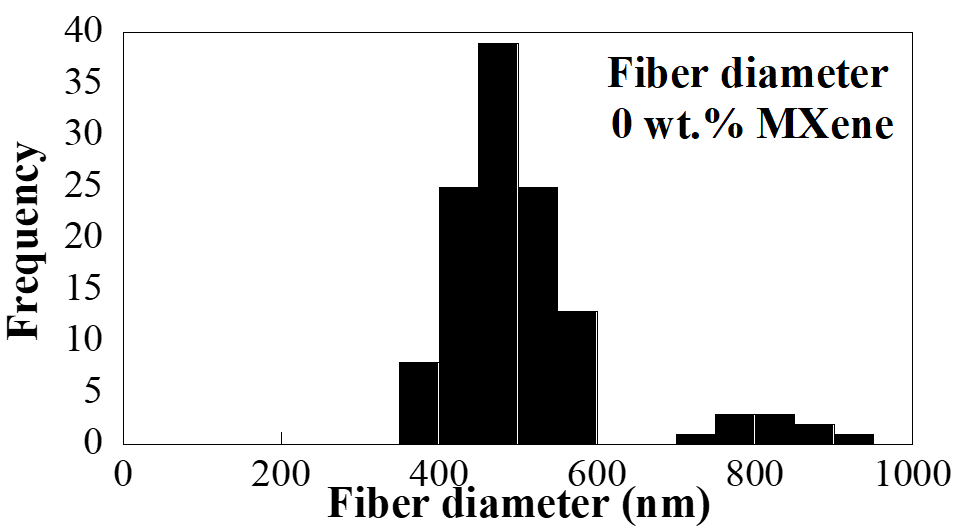  Fiber diameter = 510 ± 109 nm | N/A |
| PAN/bio-based PU  0.1 wt% MXene | 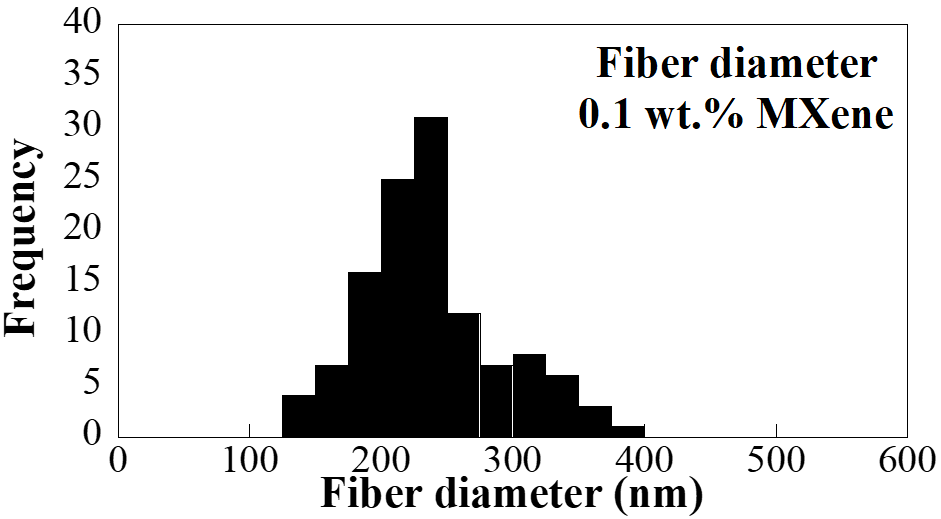  Fiber diameter = 237 ± 51 nm | 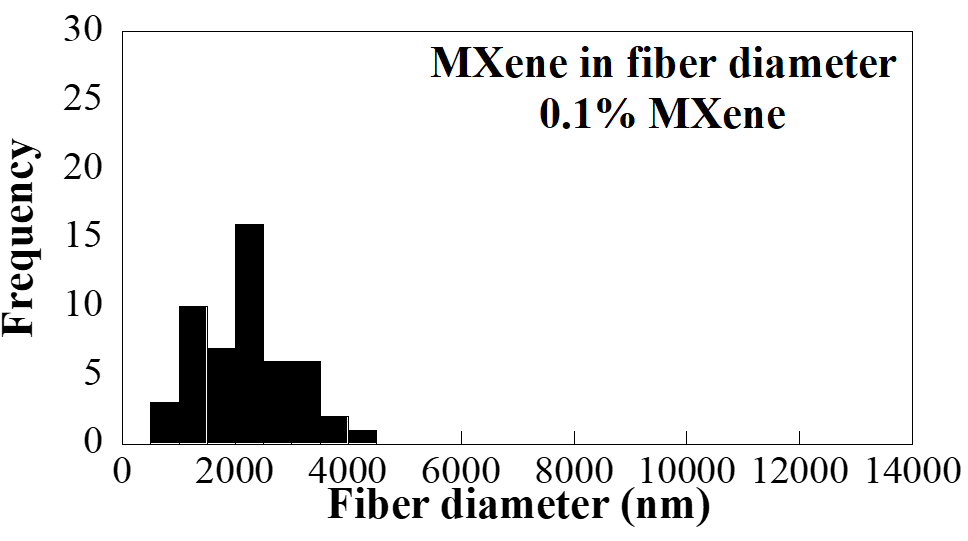  MXene in fiber diameter = 2.155 ± 0.805 μm |
| PAN/bio-based PU  1 wt% MXene | 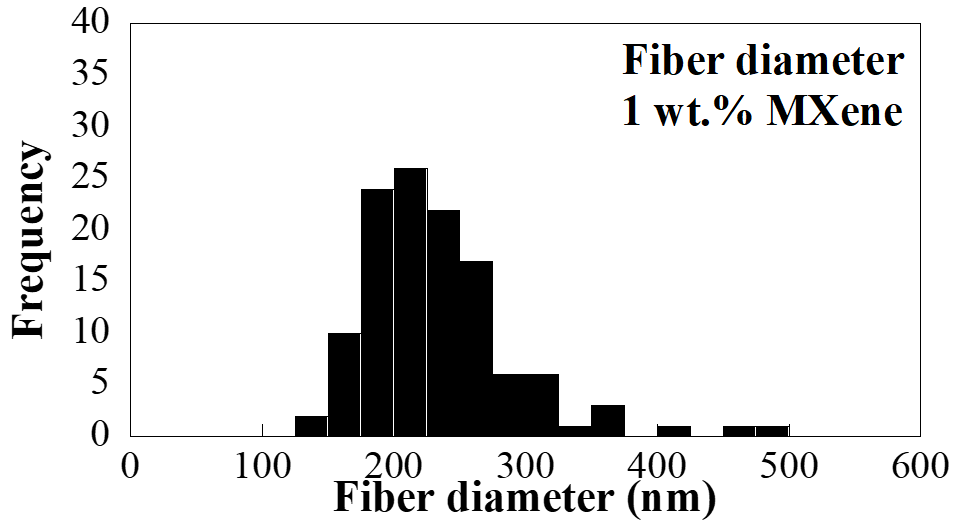  Fiber diameter = 234 ± 58 nm | 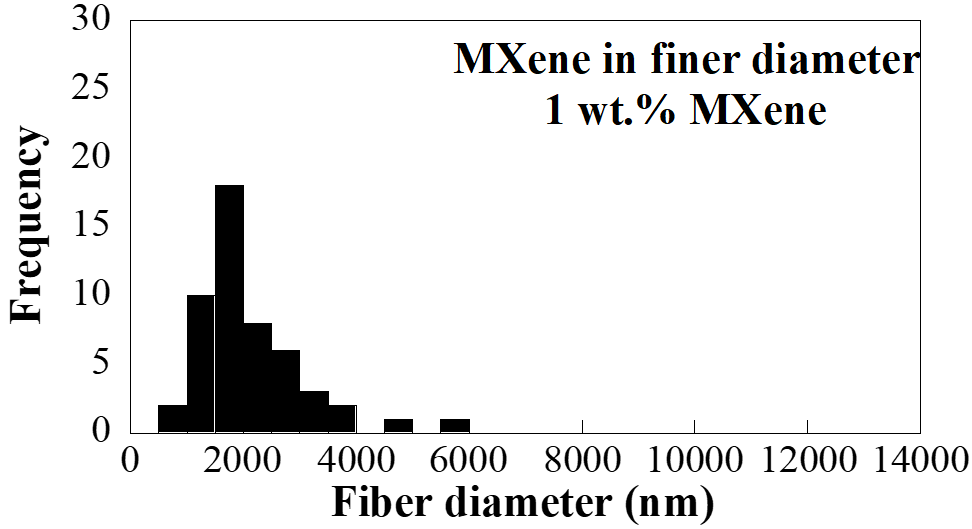  MXene in fiber diameter = 2.076 ± 0.944 μm |
| PAN/bio-based PU  5 wt% MXene | 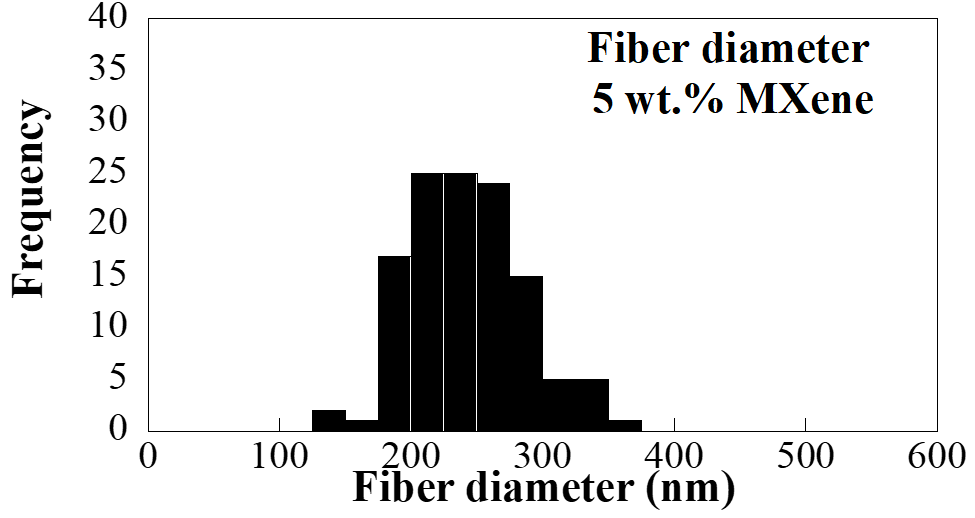  Fiber diameter = 242 ± 44 nm | 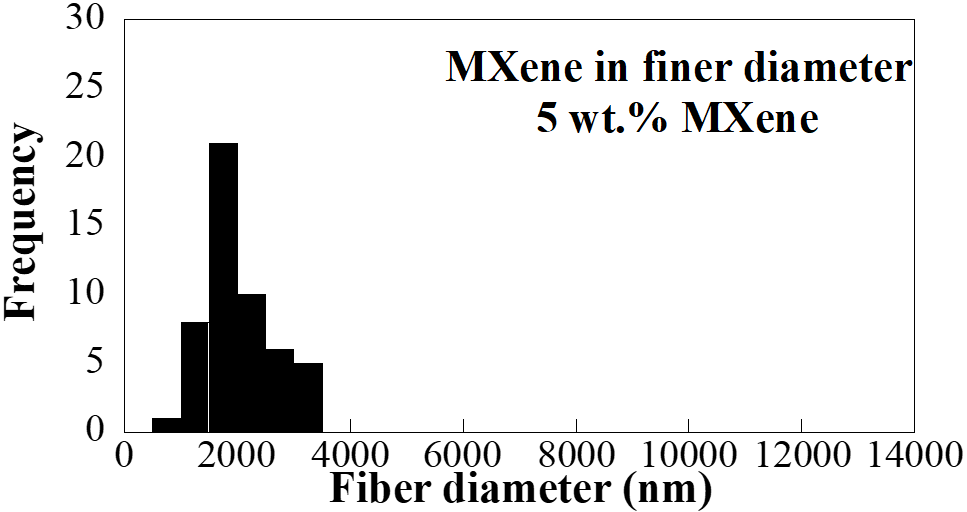  MXene in fiber diameter = 2.010 ± 0.634 μm |
| PAN/bio-based PU  7 wt% MXene | 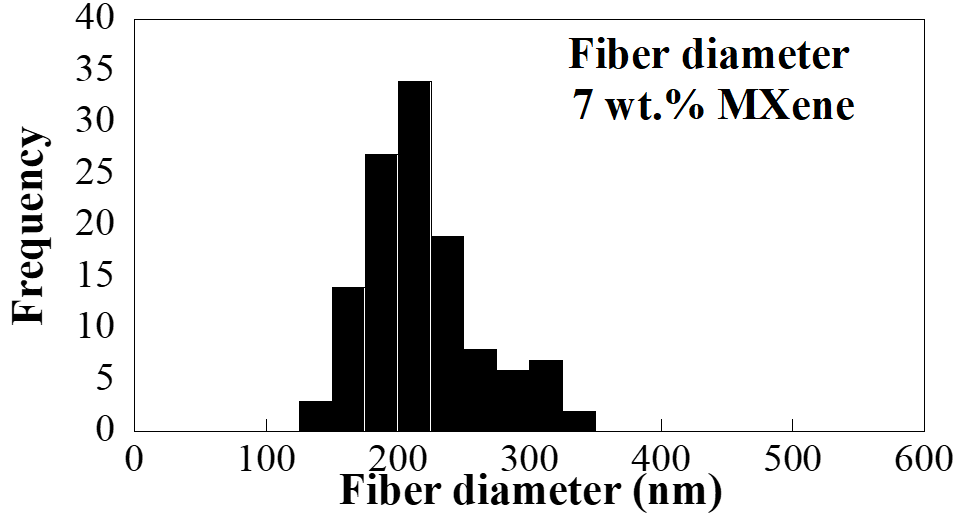  Fiber diameter = 219 ± 44 nm | 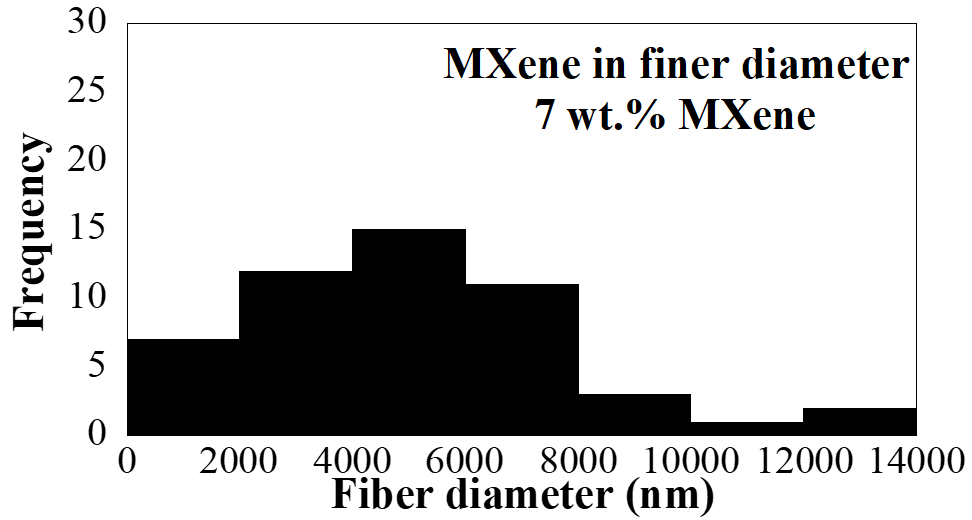  MXene in fiber diameter = 4.207 ± 2.631 μm |
| PAN/bio-based PU  10 wt% MXene | 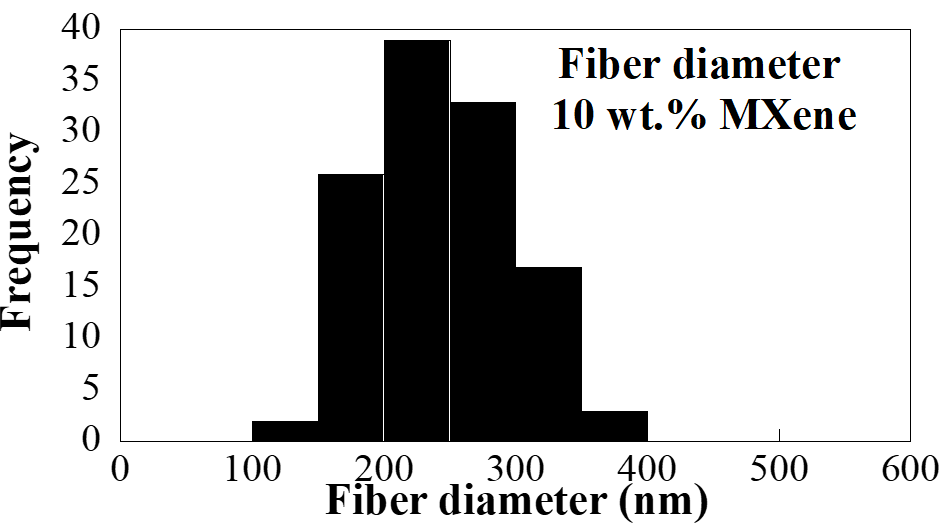  Fiber diameter = 244 ± 54 nm | 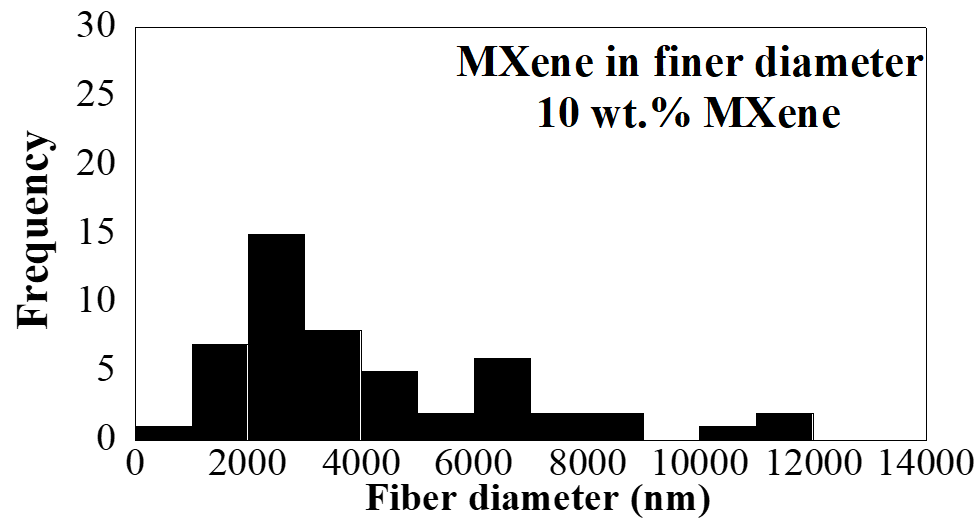  MXene in fiber diameter = 5.099 ± 2.756 μm |

**References**

1 Cao, J. et al., Oxygen defect enriched (NH_4_)_2_V_10_O_25_⋅8H_2_O nanosheets for superior

aqueous zinc-ion batteries. *Nano Energy.* **84**, 105876; 10.1016/j.nanoen.2021.105876 (2021)
